# In Vitro and In Vivo Drug Release from a Nano-Hydroxyapatite Reinforced Resorbable Nanofibrous Scaffold for Treating Female Pelvic Organ Prolapse

**DOI:** 10.3390/polym16121667

**Published:** 2024-06-12

**Authors:** Yi-Pin Chen, Tsia-Shu Lo, Yu-Han Chien, Yi-Hua Kuo, Shih-Jung Liu

**Affiliations:** 1 Department of Obstetrics and Gynecology, Keelung Chang Gung Memorial Hospital, Keelung 20401, Taiwan; 8805037@cgmh.org.tw; 2 School of Traditional Chinese Medicine, College of Medicine, Chang Gung University, Taoyuan 33302, Taiwan; 3 Department of Obstetrics and Gynecology, Chang Gung Memorial Hospital, Linkou Medical Center, Taoyuan 33305, Taiwan; 2378@cgmh.org.tw; 4 Department of Mechanical Engineering, Chang Gung University, Taoyuan 33302, Taiwan; luhan871202@gmail.com (Y.-H.C.); shelly871117@gmail.com (Y.-H.K.); 5 Department of Orthopedic Surgery, Bone and Joint Research Center, Chang Gung Memorial Hospital-Linkou, Taoyuan 33305, Taiwan

**Keywords:** resorbable meshes, drug-embedded nanofibers, sustained release

## Abstract

Pelvic prolapse stands as a substantial medical concern, notably impacting a significant segment of the population, predominantly women. This condition, characterized by the descent of pelvic organs, such as the uterus, bladder, or rectum, from their normal positions, can lead to a range of distressing symptoms, including pelvic pressure, urinary incontinence, and discomfort during intercourse. Clinical challenges abound in the treatment landscape of pelvic prolapse, stemming from its multifactorial etiology and the diverse array of symptoms experienced by affected individuals. Current treatment options, while offering relief to some extent, often fall short in addressing the full spectrum of symptoms and may pose risks of complications or recurrence. Consequently, there exists a palpable need for innovative solutions that can provide more effective, durable, and patient-tailored interventions for pelvic prolapse. We manufactured an integrated polycaprolactone (PCL) mesh, reinforced with nano-hydroxyapatite (nHA), along with drug-eluting poly(lactic-co-glycolic acid) (PLGA) nanofibers for a prolapse scaffold. This aims to offer a promising avenue for enhanced treatment outcomes and improved quality of life for individuals grappling with pelvic prolapse. Solution extrusion additive manufacturing and electrospinning methods were utilized to prepare the nHA filled PCL mesh and drug-incorporated PLGA nanofibers, respectively. The pharmaceuticals employed included metronidazole, ketorolac, bleomycin, and estrone. Properties of fabricated resorbable scaffolds were assessed. The in vitro release characteristics of various pharmaceuticals from the meshes/nanofibers were evaluated. Furthermore, the in vivo drug elution pattern was also estimated on a rat model. The empirical data show that nHA reinforced PCL mesh exhibited superior mechanical strength to virgin PCL mesh. Electrospun resorbable nanofibers possessed diameters ranging from 85 to 540 nm, and released effective metronidazole, ketorolac, bleomycin, and estradiol, respectively, for 9, 30, 3, and over 30 days in vitro. Further, the mesh/nanofiber scaffolds also liberated high drug levels at the target site for more than 28 days in vivo, while the drug concentrations in blood remained low. This discovery suggests that resorbable scaffold can serve as a viable option for treating female pelvic organ prolapse.

## 1. Introduction

Fecal pelvic organ prolapse (FPOP) stands as a pressing medical issue with profound social and economic ramifications, affecting a substantial portion of the population worldwide. Statistical data reveal a significant increase in the prevalence of FPOP cases over recent years, underscoring the urgency of effective interventions. Patients grappling with FPOP endure a myriad of challenges, ranging from physical discomfort to psychological distress, highlighting the critical need for innovative solutions [1,2,3]. Current approaches primarily involve surgical intervention combined with the grafting of artificial mesh. Non-degradable synthesis meshes composed of polypropylene (PP) have found extensive application in fortifying the pelvic floor for patients experiencing recurrent pelvic organ prolapse following native tissue repair surgery [4]. Nevertheless, the implantation of these meshes can lead to complications like mesh erosion, potentially causing issues such as tissue infections, with an occurrence frequency ranging from 7% to 25% [4]. This necessitates additional surgical procedures to eradicate the non-degradable meshes [5,6].

An optimal scaffold for therapy of FPOP should meet the following criteria: (1) possess sufficient force to maintain the pelvic floor; (2) exhibit excellent flexibility to facilitate implantation and fixation; (3) deliver precise drug or hormone levels to the intended location to achieve pain relief, infection management, and preservation of reproductive tissues, as well as stimulate the development of connective tissues to strengthen the pelvic floor; (4) degrade after completing its function and be biocompatible, ensuring that the material biodegradation process does not cause any tissue irritation [7,8].

Ongoing research endeavors aim to address these deficiencies, focusing on material innovations and treatment modalities to enhance patient outcomes [9,10,11]. Rynkevic et al. [8] investigated the potential use of electrospun nanofibers made from 2-ureido-[1H]-pyrimidin-4-one (UPy)-modified polycaprolactone (PCL) for prolapse repair. Their findings revealed that UPy-PCL nanofibrous meshes exhibited no significant weight loss or thinning during in vitro degradation. However, these meshes experienced plastic deformation and a notable change in elongation after degradation in an acidic medium. Dominguez-Robles et al. [12] developed drug-loaded thermoplastic polyurethane (TPU) meshes using fused deposition modeling additive manufacturing. They demonstrated that incorporating levofloxacin into the TPU matrix allows for the preparation of anti-infective vaginal meshes with enhanced mechanical properties compared to current PP vaginal meshes. Nevertheless, the non-degradable nature of TPU may pose a challenge in the long term. The pursuit of an optimal material for FPOP is hampered by the failure of current options to meet essential criteria, such as biocompatibility, mechanical strength, and long-term efficacy, thus delineating critical gaps in the field [5,6].

Additionally, the incorporation of therapeutic agents, such as antibiotics or anti-inflammatory drugs, within the scaffold enables targeted treatment, addressing underlying inflammation and infection, common occurrences in FPOP cases. Furthermore, the ability of the scaffold to biodegrade over time aligns with the natural healing process of the body, minimizing the risk of long-term complications associated with permanent implants. Current state-of-the-art approaches often rely on non-degradable materials or lack the capability for controlled drug release, presenting significant limitations in achieving optimal treatment outcomes. The innovation of the proposed material lies in its ability to address these critical needs simultaneously, offering a multifaceted solution that enhances therapeutic efficacy while minimizing adverse effects, thereby heralding a new era in FPOP management.

We exploited a nano-hydroxyapatite (nHA) reinforced resorbable nanofibrous scaffold for therapy of FPOP. nHA filled polycaprolactone (PCL) mesh was manufactured utilizing a solution-extrusion additive manufacturing device [13]. PCL has garnered significant attention as an implantable biomaterial due to its degradation through ester linkages hydrolysis in physiological conditions, such as those found within the human body [14]. The nHA filler, a biocompatible material renowned for its osteoconductive properties, serves as a reinforcing agent, augmenting the mechanical strength and structural integrity of the scaffold. Its integration within the material structure, strategically dispersed throughout the polymer matrix, imparts enhanced stability and biocompatibility to the final construct.

Metronidazole-, ketorolac-, bleomycin-, and estrone-embedded poly(lactic-co-glycolic acid) (PLGA) nanofibrous mats were manufactured utilizing an electrospinner [15,16,17]. PLGA stands as one of the extensively documented polymers utilized in the formulation of safe and efficient vaccine, drug, and gene delivery systems. The polymer also offers advantages over natural polymers like collagen, gelatin, or chitosan, which have drawbacks such as batch-to-batch variability, immunogenicity, and limited mechanical strength [18]. Metronidazole, an antibiotic, is extensively utilized in various medical conditions, including trichomoniasis, amebiasis, giardiasis, etc. [19], and has found various applications in obstetrics and gynecology [20]. Ketorolac has been one of the nonsteroidal anti-inflammatory drugs (NSAID) widely employed to address moderate to severe pain [21]. Bleomycin is a medication adopted to treat lung cancer, which can lead to pulmonary fibrosis, occurring upon increasing the dosage. This medication has also been reported to induce dermal fibrosis [22,23]. Estrone, an estrogen steroid hormone, serves as the principal hormone associated with the female reproductive system [24]. After fabrication, the mechanical properties of nHA reinforced mesh and drug loaded nanofibers were determined by a tensile tester. The morphology of the nanofibers was also observed using scanning electron microscopy (SEM). The in vitro discharge profiles of pharmaceuticals from the nanofibers were assessed using an elution scheme and high-performance liquid chromatography (HPLC). In addition, the in vivo elution behavior was estimated on a rat model.

In this context, the proposed innovation of a novel material emerges as a beacon of hope, poised to rectify the shortcomings of existing approaches. This groundbreaking material offers a paradigm shift in FPOP management, boasting unique features and advantages that promise to revolutionize treatment paradigms.

## 2. Materials and Method

### 2.1. Additive Manufacturing of Resorbable Mesh

**Figure 1:**
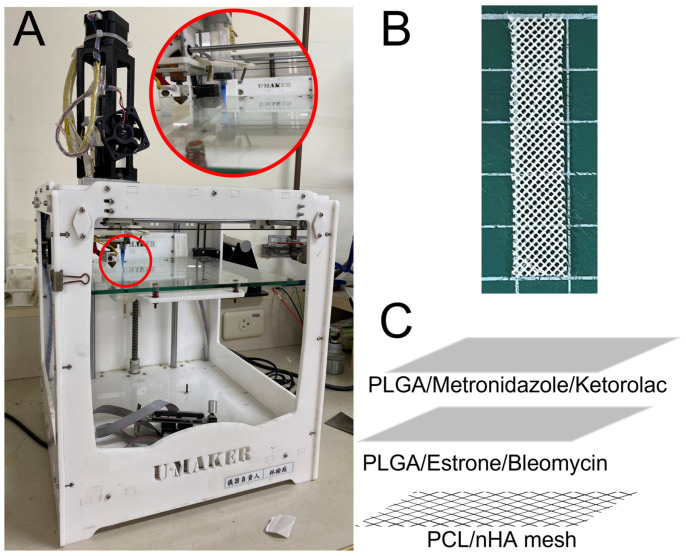
Photos of (**A**) the lab-made solution-extrusion additive manufacturing device, (**B**) additively manufactured mesh, and (**C**) schematically, the drug-eluting scaffold is composed of nHA filled PCL mesh and bi-layered drug-eluting nanofibers.

PCL (molecular weight (MW): 80 kDa) and dichloromethane (DCM) were adopted, both obtained from Sigma-Aldrich (Saint Louis, MO, USA). nHA with a size of less than 200 nm (molecular weight of 502.31 g/mol, Sigma-Aldrich) was employed as the filler. Following the design of commercially available PP mesh, biodegradable PCL mesh was fabricated employing a lab-scale solution-extrusion additive manufacturing device (Figure 1A) [13], which comprises an extrusion feeder, steering step motors, a syringe along with a delivering nozzle (inner diameter: 180 μm), a collection table, and a control port connected to a computer. PCL (2500 mg) and nHA (125 mg) were first stir mixed with DCM of 6 mL. The solution was subsequently loaded into the syringe/dispensing nozzle of the additive manufacturing device. During the additive manufacturing process, the syringe/nozzle was actuated through a computer-monitored motor. The fill density, print speed, and print orientation were set to 50%, 40 mm/s, and 60°, respectively. Once the DCM evaporates, PCL/nHA strips, approximately 0.2 mm thick, were laid on the table in successive layers. The PCL/nHA mixture was deposited on the gathering table layer-by-layer. PCL mesh, having a pore diameter of 3 mm and a thickness of 0.5 mm, was acquired on the table (Figure 1B). Meanwhile, meshes of virgin PCL were also prepared by dissolving PCL of 2.5 g in DCM of 6 mL, for comparison purpose [25,26].

### 2.2. Drug-Embedded Nanofibers

PLGA (LA:GA of 50:50, MW: 33 kDa, Sigma-Aldrich) hexafluoroisopropanol (HFIP, Sigma-Aldrich) were adopted [27,28]. Bi-layered drug-embedded nanofibers were prepared. Based on the experiences gained from our previous work on the electrospinning of PLGA nanofibers under appropriate conditions [29], PLGA (896 mg), metronidazole (112 mg), and ketorolac (112 mg) were initially stirred and blended with HFIP (4 mL), and then spun using electrospinning equipment. The rate of delivery for the solution was set at 0.7 mL/h. The voltage used was 17 kV and the travel distance from the nozzle to the gathering plate was 14 cm. To prepare the second layer, PLGA (896 mg), bleomycin (112 mg), and estrone (112 mg) were dissolved in HFIP (4 mL) and then spun. Bi-layered nanofibrous membranes were consequently produced, with a thickness of 0.21 mm (each layer measuring around 0.105 mm). After spinning, the nanofibers were combined with the printed PCL mesh (Figure 1C) into a single drug-eluting scaffold. The prepared scaffolds were placed in a chamber maintained at 40 °C for 72 h to evaporate the solvents.

### 2.3. Tensile Property

The tensile property of additively manufactured virgin PCL and nHA filled PCL meshes was assessed by a tensile testing device (Lloyd, Ametek, Berwyn, PA, USA). Specimens (2 cm × 5 cm) were cut from the meshes for the tests. The extensional rate was set at 100 mm/min and the ultimate load and deformation were recorded [30]. The tests were conducted at 25 °C, with a humidity of 60%, and three analyses were conducted (N = 3) to ensure the accuracy and reliability of the results.

Additionally, the tensile characteristic of pristine nanofibers and drug-embedded nanofibers was also evaluated. Samples of 2 cm × 5 cm were sliced from the nanofibrous membranes and measured by the Lloyd equipment (N = 3). The stretching rate was 100 mm/min [27,28].

### 2.4. Scanning Electron Microscope (SEM) Observation

The microscopic structure of printed mesh and spun nanofibers was appraised by utilizing SEM after they were coated with gold. The dimensional distribution of electrospun fibers was determined by analyzing 100 randomly selected fibers (N = 3) using a commercial ImageJ (version 1.49) code provided by the National Institutes of Health, Bethesda, MD, USA.

### 2.5. Wetting Angle

To assess hydrophilicity, wetting angle of pristine PLGA and drug-embedded PLGA nanofibrous membranes was measured. Distilled water was gently deposited on the nanofiber surface (1 cm × 1 cm) and analyzed employing a video monitor. The measurement was conducted in triplicate (N = 3).

### 2.6. Fourier Transform Infrared Spectroscopy (FTIR)

Fourier transform infrared spectroscopy (FTIR) analyses were performed to assess the nanofibers’ composition and verify the presence of all introduced pharmaceuticals. To acquire the spectra of the nanofibers charged with the drug, Fourier transform infrared spectroscopy (FTIR) was completed using the Bruker Tensor 27 spectrometer (Billerica, MA, USA). The analysis was conducted at a resolution of 4 cm^−1^ in the absorption mode, with a total of 32 scans. The nanofibrous specimen was pressed into KBr discs, with the spectra recorded within the span of 400–4000 cm^−1^ (N = 3).

### 2.7. Differential Scanning Calorimetry Assessment

Scanning calorimetry (DSC) assays were conducted to evaluate the composition of the nanofibers and confirm the inclusion of added pharmaceuticals. The thermal characteristics of pristine PLGA nanofibers and drug-embedded PLGA nanofibers were examined through DSC using TA Instruments equipment located in New Castle, DE, USA. The polymeric specimens underwent scanning from 30 °C to 300 °C, with a constant heating speed of 10 °C/min (N = 3).

### 2.8. In Vitro Drug Release

The discharge characteristic of metronidazole, ketorolac, bleomycin, and estrone from the drug-embedded nanofibrous membranes was investigated. The nanofibrous sample (approximately 1 cm × 1.5 cm) was filled into a glass tube containing phosphate-buffered saline (PBS) of 1 mL (N = 3). The tube was then placed in an isothermal chamber at 37 °C for 24 h, before the solution was gathered and analyzed. New PBS of 1 mL was added to the tube for the subsequent 24 h interval, and this procedure was repeated for a total of 30 days.

The pharmaceutical concentrations in the gathered solutions were determined utilizing HPLC, conducted on a Hitachi L-2200R multi-solvent delivery system based in Tokyo, Japan. For metronidazole [31], the mobile phase consisted of acetonitrile:PBS in a ratio of 30:70 (*v*/*v*) at pH 7.0. The assay parameters included a wavelength of 319 nm and a flow rate of 1.0 mL/min. An Inertsil C18 column (250 × 4.6 mm × 5 mm) from Supelco (Sigma-Aldrich) was employed, with a retention time ranging from 3.5 to 4.3 min. To assay ketorolac, an Ascentic C18 (250 × 4.6 mm × 5 mm) column (Supelco, Sigma-Aldrich) was employed. The mobile phase included acetonitrile and 5 mM ammonium acetate (pH 3.5) in a ratio of 60:40 (*v*/*v*) [32]. Absorbance was measured at a wavelength of 306 nm, with a flow rate of 1.0 mL/min. The retention time was set at 2.5 min.

Additionally, the distilled water:acetonitrile:acetic acid (with 0.0085 M sodium heptanesulfonate) at a ratio of 70:25:5 (*v*/*v*/*v*) was adopted as the mobile phase for bleomycin [33]. Absorbance was measured at a wavelength of 295 nm, with a flow rate set at 1.0 mL/min. An Ascentic C18 (150 × 4.6 mm × 5 mm) column (Waters, MA, USA) was adopted for the analysis. The duration of retention was set at 1.6 min. Finally, to evaluate estrone, a Mightysil RP-18 GP 250-4.6 (5 µm) column (Kanto Chemical, Tokyo, Japan) was utilized. The mobile phase contained acetonitrile:PBS (35:65, *v*/*v*) [34]. Absorbance was measured at a wavelength of 280 nm, with a flow rate set at 1.0 mL/min. The duration of retention was set at 6.8 min. All experiments were completed in triplicate (N = 3).

### 2.9. In Vivo Drug Elution

**Figure 2:**
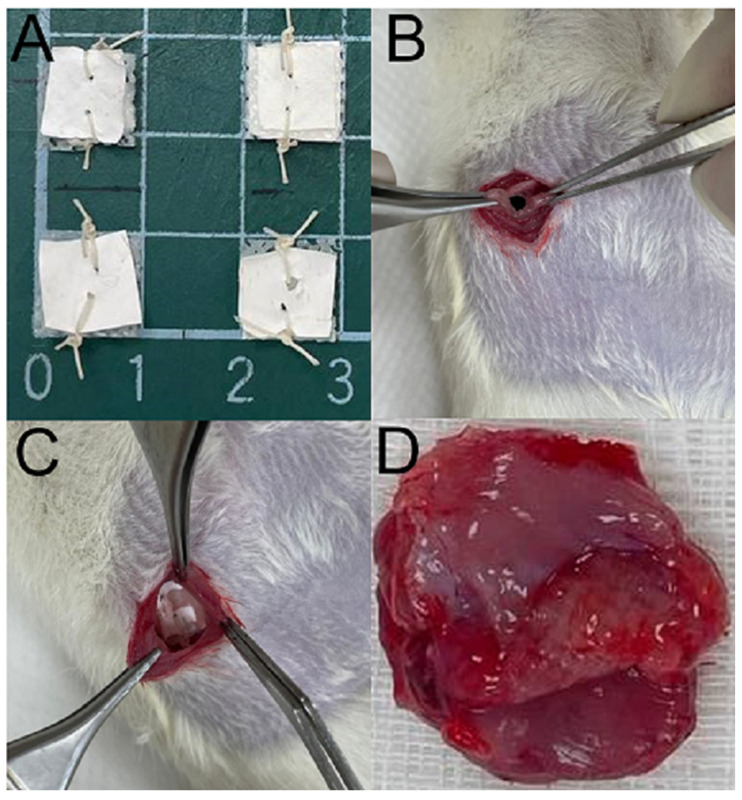
PCL mesh implantation and retrieval. (**A**) Integration of nHA filled PCL meshes and drug-loaded PLGA nanofibers via sutures, (**B**) exposed peritoneal herniation, (**C**) placement of mesh/nanofibers on peritoneal defect, (**D**) retrieved mesh/nanofibers with peritoneum at 28 days post-implantation.

Sprague-Dawley rats (approximately 300 g) received treatment and care under the supervision of a licensed veterinarian, following the regulations of the National Institute of Health of Taiwan. The entire procedures involving animals acquired approval from the Institutional Animal Care and Use Committee of Chang Gung Memorial Hospital (IACUC: CGMH2018121905). The animals were initially anesthetized with isoflurane. Before implantation, nHA filled PCL meshes and drug-loaded PLGA nanofibers were integrated via sutures (Figure 2A). A 4 cm cut was made in the lower abdomen of each rat (Figure 2B). Following the implantation of resorbable nanofibrous scaffold (3 cm × 1 cm × 5 mm in dimension) (Figure 2C), the wound was sealed using 3-0 Vicryl sutures (Ethicon Inc., Bridgewater, NJ, USA).

To assess the in vivo release pattern of drug-eluting scaffolds, tissues surrounding the scaffolds were collected on days 1, 3, 7, 14, and 28 (Figure 2D) after implantation using an operational process the same as that employed during the implantation. The pharmaceutical level in the gathered specimens was then determined by HPLC. Additionally, the obtained specimen was stained with hematoxylin and eosin (H&E) and Masson’s trichrome staining, and examined under a microscope.

## 3. Results

### 3.1. Characterization of Drug-Embedded Mesh/Nanofibers

**Figure 3:**
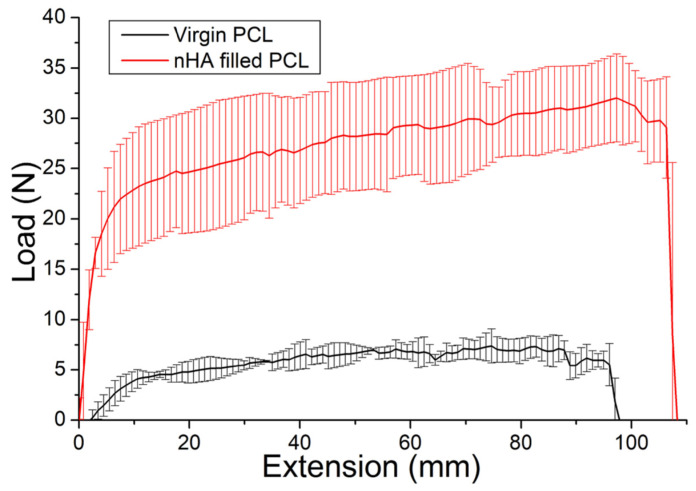
Tensile properties of additively manufactured virgin polycaprolactone (PCL) meshes and nHA filled PCL meshes.

Figure 3 displays the tensile test curves of manufactured mesh. The nHA filled PCL meshes exhibited superior strength to that of virgin PCL meshes, demonstrating the capability of nHA in reinforcing the PCL meshes’ function as supporting scaffolds for pelvic floor repair. Additionally, the toughness of nHA filled PCL mesh and virgin PCL was 3.12 J and 0.64 J, respectively. Clearly, the nHA filled mesh exhibited greater toughness than the unfilled mesh. Compared to the unfilled PCL mesh, the nHA-filled mesh exhibited greater deviations among different test trials. This suggests that the dispersion of nHA within the PCL matrix may require optimization during the mixing and printing processes. Enhanced uniformity in nHA distribution is likely necessary to achieve consistent material properties and performance across all trials.

**Figure 4:**
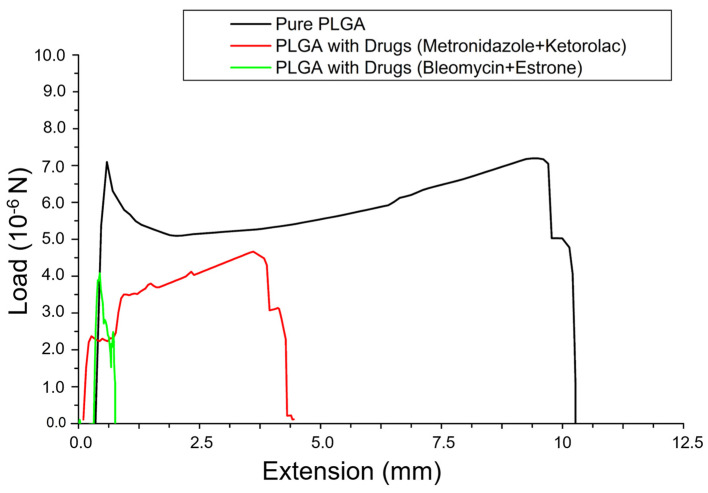
Stress–strain curves of virgin poly (lactic-co-glycolic acid) (PLGA) nanofibers and drug-loaded PLGA nanofibers.

The mechanical properties of the electrospun nanofibrous membranes were also measured. The experimental results in Figure 4 suggest that virgin PLGA nanofibers had greater tensile strength than the drug-loaded nanofibers. Additionally, the virgin nanofibers exhibited greater elongation at breakage compared to the drug-eluting nanofibers. The inclusion of pharmaceuticals in the nanofibers reduces the polymer content, which provides the major resistance to external tensile force. Consequently, the measured mechanical properties decreased accordingly.

**Figure 5:**
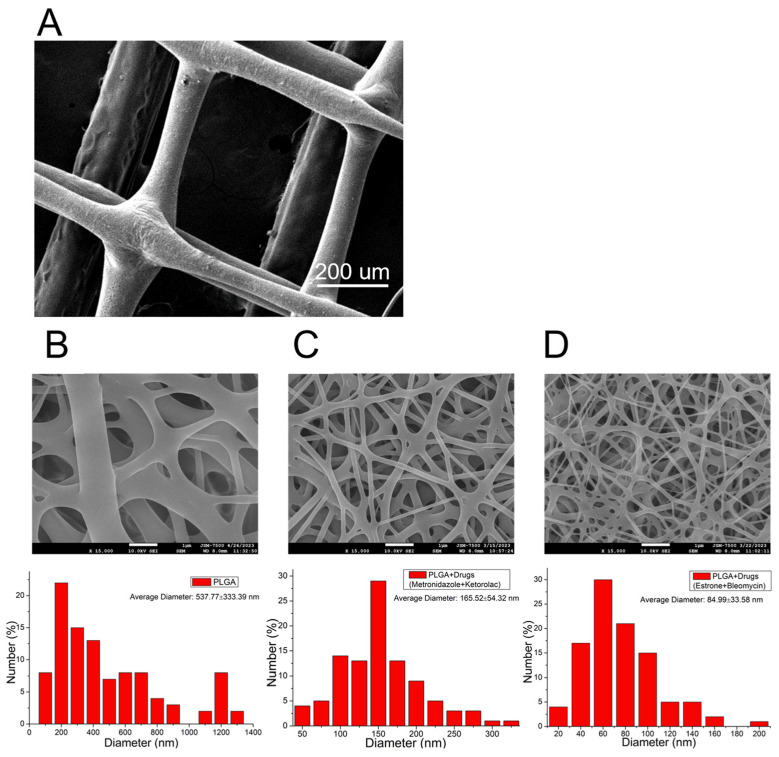
Scanning electron microscopy (SEM) images and fiber diameter distribution of (**A**) polycaprolactone (PCL) mesh, (**B**) virgin poly(lactic-co-glycolic acid) (PLGA) nanofibers, (**C**) metronidazole/ketorolac/PLGA nanofibers, and (**D**) bleomycin/estrone/PLGA nanofibers. Spun virgin PLGA nanofibers (537.8 ± 333.4 nm) exhibited superior size distribution to those of metronidazole/ketorolac/PLGA nanofibers (165.5 ± 54.3 nm) and bleomycin/estrone/PLGA nanofibers (84.9 ± 33.6 nm).

Figure 5A shows the SEM image of the printed PCL mesh. With the addition of nHA, some rough surfaces are noted. Figure 5B–D display the structures of pristine PLGA nanofibers, metronidazole- and ketorolac-incorporated PLGA nanofibers, and bleomycin- and estrone-embedded PLGA nanofibers, respectively. Spun virgin PLGA nanofibers (537.8 ± 333.4 nm) exhibited superior size distribution to those of metronidazole/ketorolac/PLGA nanofibers (165.5 ± 54.3 nm) and bleomycin/estrone/PLGA nanofibers (84.9 ± 33.6 nm). During the electrospinning process, an external electric force is applied to stretch the polymeric mixture. Polymers serve as the primary constituent to oppose the exterior stretching force. In comparison to the pristine nanofibers, the integration of pharmaceuticals resulted in a reduction in the percentage of polymers within the nanofibers. The nanofibers became more susceptible to extension by the external force, resulting in a corresponding decrease in their diameters.

**Figure 6:**
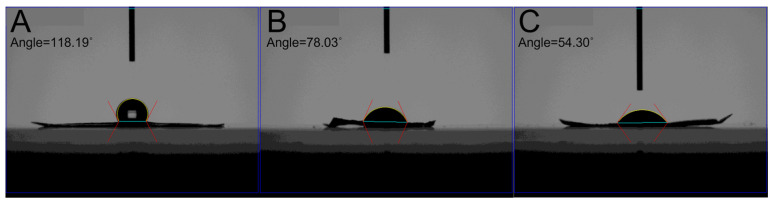
Water contact angles of (**A**) virgin poly (lactic-co-glycolic acid) (PLGA) nanofibers, 118.2°, (**B**) metronidazole and ketorolac loaded PLGA nanofibers, 78.0°, (**C**) bleomycin and estrone embedded PLGA nanofibers, 54.3°.

Figure 6 shows the measured contact angles of virgin PLGA, metronidazole/ketorolac/PLGA, and bleomycin/estrone/PLGA nanofibers. The measured water contact angle for pristine nanofibers was 118.2 degrees, whereas the contact angles were 78.0 degrees and 54.3 degrees for the metronidazole/ketorolac/PLGA and bleomycin/estrone/PLGA nanofibers, respectively. Despite that pure PLGA nanofibers exhibited hydrophobic characteristics, the inclusion of water-soluble pharmaceuticals significantly increased the hydrophilicity of the electrospun nanofibers.

**Figure 7:**
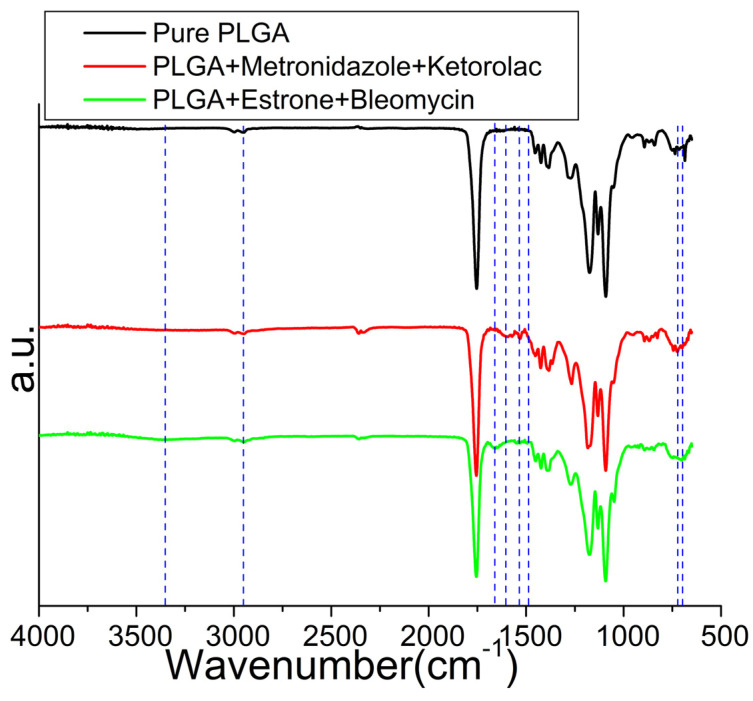
Fourier transform infrared spectroscopy (FTIR) spectra of metronidazole and ketorolac loaded PLGA nanofibers, and bleomycin and estrone embedded PLGA nanofibers.

Figure 7 illustrates the FTIR spectra of pristine PLGA nanofibers and drug-embedded PLGA nanofibers. The vibration peak at 702 cm^−1^ was promoted owing to the C–H bond of incorporated drugs. The fresh peaks at 1593 cm^−1^ and 1530 cm^−1^ were primarily attributed to the C=C bonds of metronidazole [35] and ketorolac [36]. Furthermore, the newly observed peak at 1658 cm^−1^ was associated with the C=N bond of bleomycin [37], and the additional peak at 702 cm^−1^ was intensified by the C–H bond of incorporated estrone [38]. Additionally, the peaks at around 1400 cm^−1^ and 3000 cm^−1^, attributed to OH bonds, were enhanced owing to the inclusion of the pharmaceuticals.

**Figure 8:**
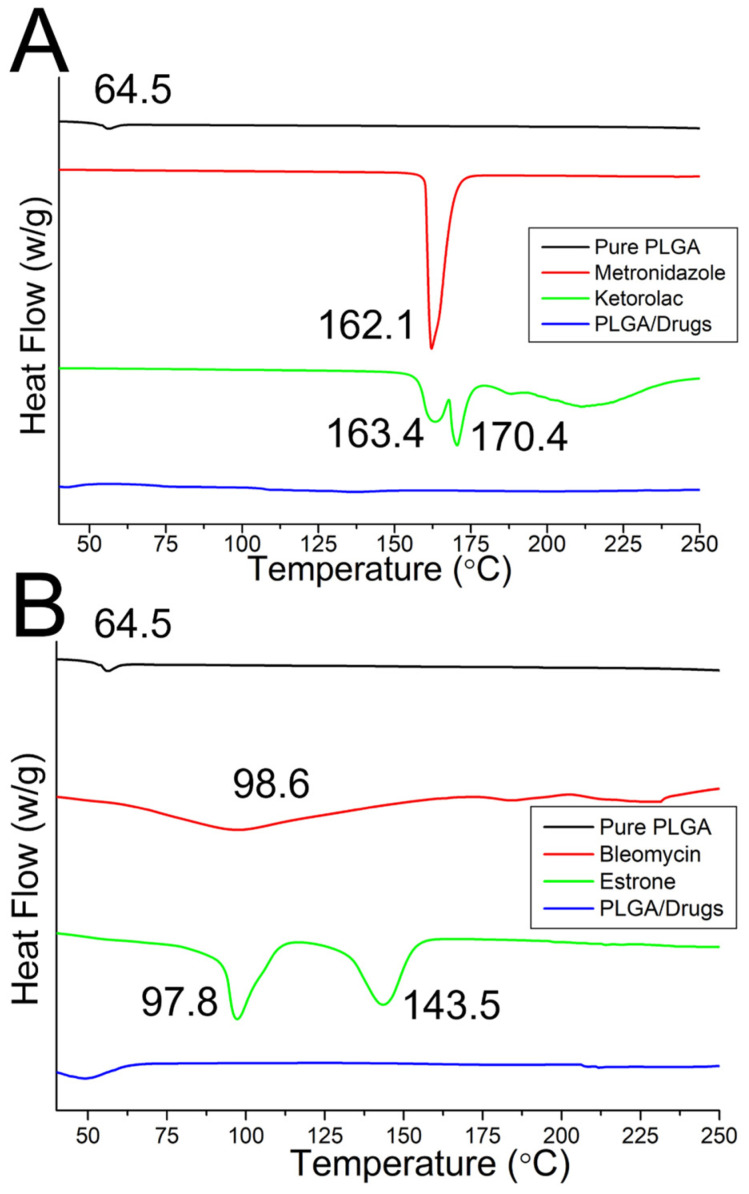
Fourier transform infrared spectroscopy (FTIR) spectra, (**A**) metronidazole and ketorolac loaded PLGA nanofibers, (**B**) bleomycin and estrone embedded PLGA nanofibers.

Figure 8A depicts the thermograms of virgin PLGA and metronidazole/ketorolac-embedded PLGA nanofibers. The exothermal peaks at 162.1 °C for metronidazole [39] and at 163.4 °C and 170.4 °C [40] for ketorolac diminished after mixing with PLGA. Further, Figure 8B shows the DSC curves for both virgin PLGA and bleomycin and estrone loaded PLGA nanofibers. Again, the exothermal peaks at 98.6 °C for bleomycin [41] and at 97.8 °C and 143.5 °C [42] for estrone could not be found after mixing with PLGA.

All these assays affirmed the successful embedding of pharmaceuticals in the PLGA nanofibers.

### 3.2. In Vitro and In Vivo Drug Releases

**Figure 9:**
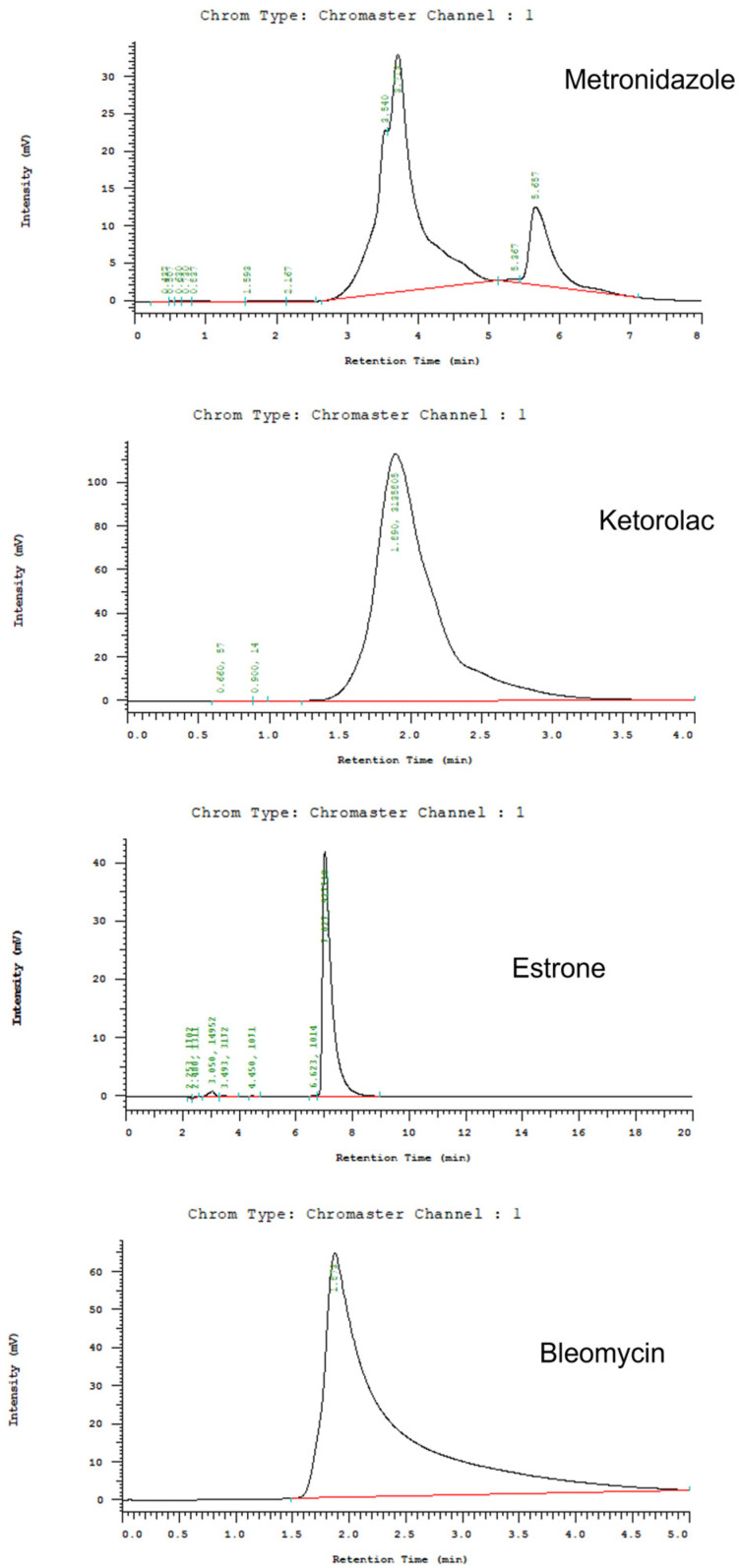
HPLC chromatograms of the pharmaceuticals.

**Figure 10:**
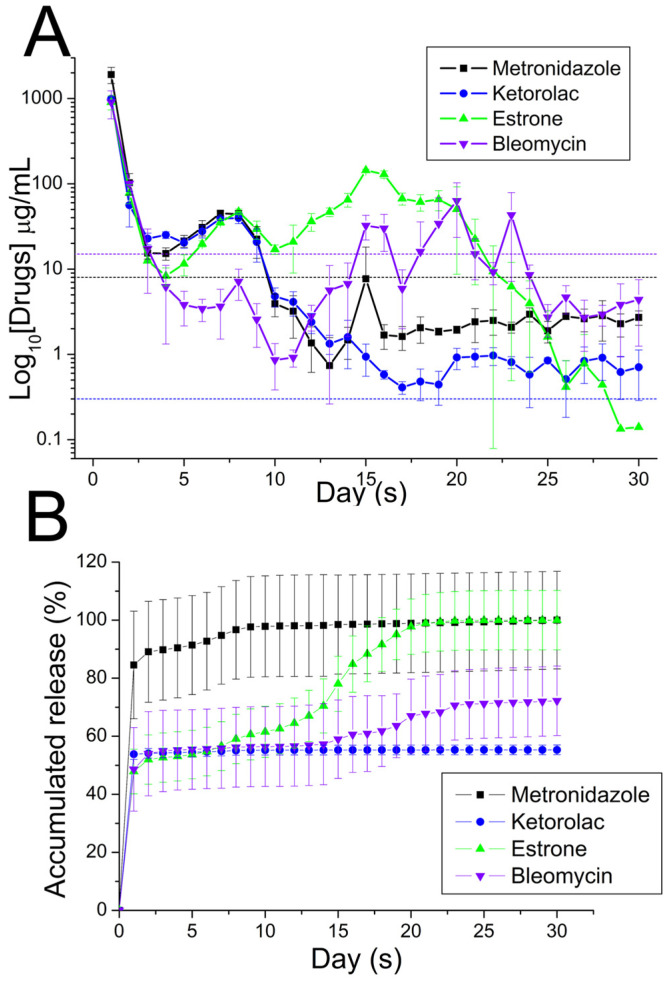
In vitro (**A**) daily, (**B**) cumulative release of pharmaceuticals from the nanofibers. The minimum inhibitory concentrations (MICs) of metronidazole, ketorolac, and bleomycin were 8, 0.03, and 15 μg/mL, respectively. Meanwhile, the minimum therapeutic concentration (MTC) of estrone was 1.36 pg/mL, which is too low to be shown in the upper figure.

Figure 9 displays the HPLC chromatograms of the pharmaceuticals employed in this work. Figure 10 depicts the daily and accumulative discharge profiles of the pharmaceuticals from the nanofibers in vitro. Generally, all pharmaceuticals displayed a tri-phase discharge pattern, i.e., a primary peak at day 1, minor peak releases at various days. This is followed by a consistently and progressively decreasing release. Metronidazole showed the minor peaks at days 7 and 15. Ketorolac displays only one minor peak at day 7, while bleomycin showed minor peaks at 8, 15, 20, and 23 days. Meanwhile, the release of estrone showed tiny bursts at days 7 and 15. Furthermore, the spun nanofibrous membranes consistently released effective metronidazole (above the minimum inhibitory concentration [43]) for a duration of 9 days, and discharged effective bleomycin and ketorolac (above the minimum therapeutic concentrations [44,45]) for 3 and 30 days, respectively. The nanofibrous membrane additionally demonstrated continuous discharge of estrone [46], spanning a period of more than 30 days.

**Figure 11:**
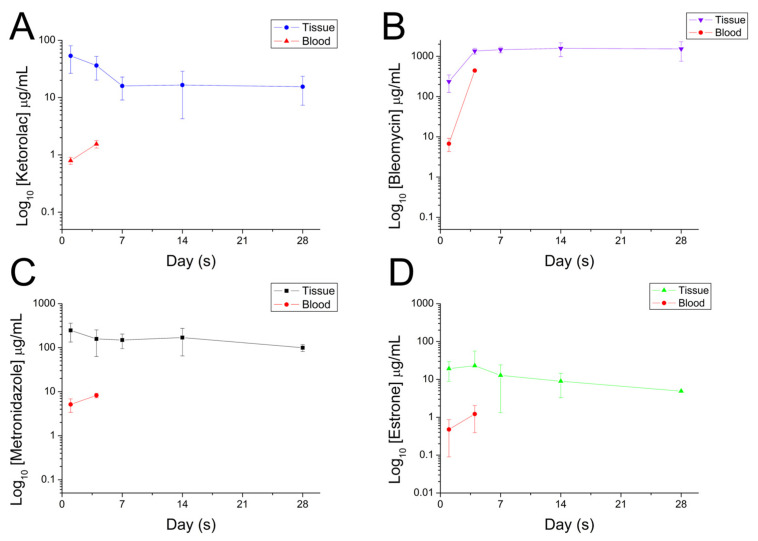
In vivo release of (**A**) ketorolac, (**B**) bleomycin, (**C**) metronidazole, (**D**) estrone from the resorbable nanofibrous prolapse meshes.

**Figure 12:**
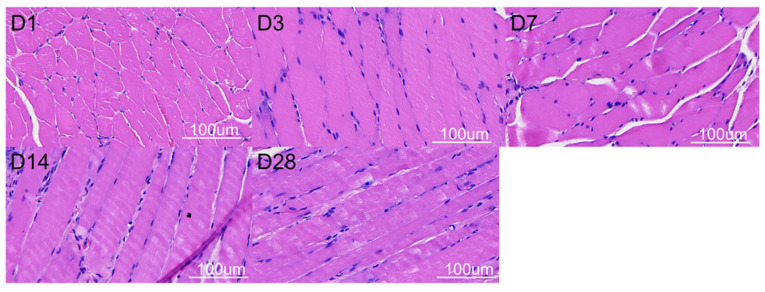
Hematoxylin and eosin staining of tissues. (D1 represents as day 1; D3 represents as day 3; D7 represents as day 7; D14 represents as day 14; D28 represents as day 28) (scale bar: 100 μm).

**Figure 13:**
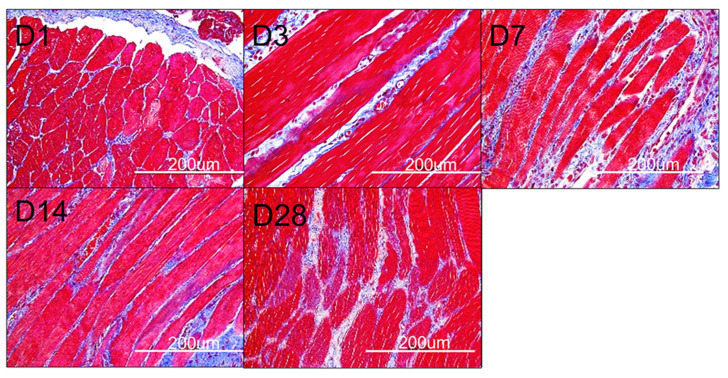
Histological images (400×) with Masson’s trichrome stain post-implantation. Red area: muscle fiber, blue area: collagen fiber. (D1 represents as day 1; D3 represents as day 3; D7 represents as day 7; D14 represents as day 14; D28 represents as day 28) (scale bar: 200 μm).

Figure 11 displays the in vivo discharge characteristic of pharmaceuticals from the resorbable nanofibrous scaffolds. The scaffolds discharged high drug concentrations at local tissues for over 28 days. Meanwhile, the pharmaceutical levels in the blood were relatively low at days 1 and 3. Figure 12 displays images of H&E staining at various days post-implantation, while Figure 13 shows histological images with Masson’s trichrome staining at different days post-implantation. Fibrosis variations were observed following the implantation of the drug-eluting PCL mesh. Additionally, Masson’s trichrome staining confirmed a significant increase in collagen fibers.

## 4. Discussion

In our study, we employed additive manufacturing and electrospinning techniques to fabricate resorbable scaffolds embedded with multiple drugs. These scaffolds feature PCL meshes as the backbone to enhance mechanical strength and PLGA nanofibers as the drug delivery vehicle, with the aim of providing therapy for FPOP. PCL is a degradable and compatible synthetic polymer belonging to the class of aliphatic polyester. The polymer’s unique combination of properties, such as biodegradability, biocompatibility, low melting point, and flexibility and durability, makes it a valuable material in various fields, particularly in drug delivery and tissue engineering [47]. Our past study on PCL employing cell culture shows that the material exhibited good cell biocompatibility [48]. Additionally, the slow degradation rate of PCL typically leads to a complete resorption period of 2 to 3 years. The results of our recent work suggest that the maximum tensile strength of PCL did not show an obvious reduction over time after being submerged in a buffered solution for four months [49].

Additive manufacturing is a manufacturing process that builds objects layer by layer using computer-aided design (CAD) data. The technique has made significant inroads in the field of medicine and healthcare due to its unique capabilities and advantages. Key medical applications include custom implants and prosthetics, tissue and organ bioprinting, dental restorations, and personal medications etc. [50,51].

In contrast, PLGA is a biodegradable and biocompatible copolymer derived from the combination of two naturally occurring compounds, lactic acid and glycolic acid. PLGA is a widely used synthetic polymer in the field of biomedicine, pharmaceuticals, and tissue engineering due to its favorable properties such as biodegradability, biocompatibility, and tunable degradation rate [52]. Electrospinning is a nanofiber fabrication technique that utilizes an electrostatic field to produce ultrafine fibers, in the range of tens to hundreds of nanometers, from polymer solutions or melts. This ultrafine scale is valuable in distinct applications, such as tissue engineering, filtration, and drug delivery, where controlling material properties at the nanoscale is crucial [53,54].

Extensively investigated for tissue engineering applications, electrospun PLGA fibers possess a distinctive micro-/nanofibrous morphology that closely mimics the extracellular matrix. Spun nanofibers exhibit high porosity, a significant aspect ratio, and robust strength, facilitating improved tissue proliferation. These attributes endow nanofibers with considerable applications for drug delivery and tissue engineering. A hydrophilic surface promotes cell adhesion and proliferation, crucial for tissue engineering applications. It also aids in controlled drug release by facilitating the absorption of water, allowing for sustained release over time. Mukherjee and colleagues [55] suggested that nanofibrous meshes offer a unique surface structure capable of retaining therapeutic cells for an extended period of up to six weeks, promoting significant infiltration by anti-inflammatory macrophages from the host. Achieving effective cell attachment to the scaffold is critical for success. Nevertheless, a challenge persists; the adoption of highly hydrophobic scaffold materials may lead to suboptimal cell colonization. Incorporating pharmaceuticals into the nanofibers enhances their hydrophilic properties [56], thereby fostering the growth of connective tissues for FPOP repair. PLGA nanofibers with incorporated drugs also exhibited exceptional flexibility and extensibility, making them well-suited for functions in pelvic floor repair. This characteristic facilitates tissue contraction in the recovery process. Furthermore, nanofibers produced through electrospinning and embedded with pharmaceuticals offer customizable and sustained drug release. Localized pharmaceutical transport ensures elevated and sustained drug levels in the designated tissue, minimizing systemic doses and associated risks of hypoglycemia.

In general, the release of drugs from a resorbable device containing pharmaceuticals follows three separate phases: burst release, diffusion-governed discharge, and degradation-governed elution [57]. In the electrospinning procedure, a significant portion of charged drugs is encased within the polymeric matrix volume. However, a few drugs may be situated on the surface of the fibrous membrane, resulting in burst release. Following the initial peak release, the drug liberation is regulated through diffusion and polymer degradation. Minor peak drug discharges could then be observed for the incorporated pharmaceuticals; thereafter, the release profiles diminished progressively. The empirical findings substantiated that the produced nanofibers release effective ketorolac and estrone (above the minimum therapeutic concentration) for a period of 30 days. This is advantageous for pain control [58] and can aid in alleviating vaginal symptoms of menopause, including vaginal dryness, burning, and itching [59]. However, the nanofibrous membrane only sustainably discharges efficient metronidazole and bleomycin (above the minimum inhibitory concentration) for 9 and 3 days in vitro, respectively. Fortunately, the electrospun nanofibers released elevated drug levels for over 28 days in vivo, offering advantages as an effective scaffold for FPOP repair.

Kao et al. [60] developed degradable anti-adhesive PLGA nanofibrous membranes incorporating vancomycin/ceftazidime, ketorolac, and human epidermal growth factor (hEGF). Their findings revealed that these membranes released high concentrations of vancomycin/ceftazidime, ketorolac, and hEGF in vitro for over 30, 24, and 27 days, respectively. Moreover, they demonstrated that the anti-adhesive nanofibers provided postoperative pain relief and infection control, while also promoting the healing of surgical wounds in a rat model. Tugzu-Demiroz and colleagues [61] employed electrospinning to create metronidazole-loaded nanofibers and proposed them as a potent pharmaceutical delivery system for addressing bacterial vaginosis. In a related investigation, Srithep et al. [62] formulated electrospun nanofiber mats incorporating metronidazole for addressing periodontal disease. They observed an entrapment efficiency of metronidazole within the range of 82% to 99%, featuring an initial release of no less than 30% of the drug, followed by sustained release spanning 7 days. To prolong the duration of sustained drug release from PLGA mats, potential strategies encompass higher pharmaceutical loading, an augmented polymer/drug ratio, the utilization of greater molecular-weight polymers, and/or the establishment of a strongly oriented nanofibrous structure. On the other hand, bleomycin is a medication used in the treatment of cancer, specifically as a chemotherapeutic agent [63]. It is part of a class of drugs known as antitumor antibiotics, which also induce tissue fibrosis. However, bleomycin is associated with potentially severe side effects, including pulmonary toxicity and skin reactions. Utilizing resorbable nanofibers, we can achieve localized delivery of medication to the pelvic floor. This approach enables the delivery of high drug concentrations directly to the target tissue, promoting tissue fibrosis and strengthening the pelvic floor. Meanwhile, the systemic drug level in blood is low, avoiding the risk of potential lung toxicity.

There were constraints within this initial study. We utilized a rat model to assess the effectiveness of drug-loaded scaffolds. The animal model employed was a healthy model rather than a diseased model. In addition, the applicability of the current discoveries to individuals with pelvic organ prolapse is uncertain and necessitates additional investigation. Considering the mechanical support that the scaffold should provide, some long-term mechanical tests and live ultrasounds could be included to comprehensively evaluate the efficacy of the scaffold. Long-term degradation tests and cellular assessments of printed mesh and spun nanofibers may be needed to confirm the non-toxicity of the scaffolds. Further experiments are also required to validate the potential of bleomycin in promoting tissue fibrosis for pelvic floor repair. These areas become the focus of forthcoming research endeavors.

## 5. Conclusions

In this investigation, degradable nHA-filled PCL mesh/drug-eluting PLGA nanofibrous scaffolds were developed to replicate the microstructure of the natural extracellular matrix found in various connective tissues, created through additive manufacturing and electrospinning techniques. The proposed scaffold holds promise in mitigating potential complications associated with the treatment of FPOP while offering significant enhancements to current therapeutic approaches. One potential complication that the novel material may alleviate is the risk of mesh-related complications, commonly observed with traditional PP meshes, such as erosion, infection, and chronic pain. By utilizing a resorbable PCL mesh reinforced with nHA, augmented by drug-eluting PLGA nanofibers, the proposed scaffold offers improved biocompatibility and reduced risk of adverse reactions. Additionally, the enhanced mechanical properties of the scaffold provide robust support to pelvic organs, minimizing the likelihood of recurrence and prolapse. Furthermore, the controlled release of therapeutic agents from the PLGA nanofibers offers targeted treatment, addressing inflammation and promoting tissue regeneration, thereby fostering better long-term outcomes for individuals grappling with FPOP.

## Data Availability

The data used to support the findings of this study are included within the article.
